# Croup

**Published:** 1847-10

**Authors:** 


13. *Croup.*—Dr. Blakeman of New York, in two cases of Croup, cauterized the Larynx with a solution of Nitrate of Silver. In the first case, "a child was two years old, very fat and large of his age, and of leuco-phlegmatic temperament;" the symptoms were well marked, the "skin hot and dry, pulse quick, great restlessness, laborious breathing, and the hoarse barking or crowing sound peculiar to Croup." Emetics were freely given, and active vomiting ensued, but none of "the peculiar heavy glairy substance, which is the secretion of this specific inflammation," was ejected. Copions discharges from the bowels were meanwhile obtained by the calomel which had been administered, but "the remedies having done no good, and the symptoms of suffocation becoming alarming, it was resolved to try the effect of cauterizing the larynx with a sol. Nit. Arg. ʒ j to ℥ j of water. The application was somewhat difficult, and the dyspnœa very great. A quantity of the thick tenacious substance was brought away by the sponge, &c., and a large quantity by the vomiting which followed. Ten minutes afterwards the operation was repeated with similar effects, and the disease appeared to be arrested. Five and a half hours after the first application, he "had improved in all the symptoms: breathing decidedly better, the barking sound heard only at intervals, and he had asked for drink;" the sponge was again used, and with similar results: and, after the cessation of the subsequent vomiting, the child fell asleep. The following day a slight hoarseness remained, for which he required no further treatment. In the second case, a boy six years old, in whom the febrile symptoms were well marked, Dr. B. "determined that the remedy used last in the former case, should be the first in this, and made two applications of the sponge, with a solution of the same strength as before employed. Some tough phlegm came away on the sponge, and free vomiting followed, which relieved the patient so that he fell asleep."—*N. Y. Med. & Surg. Reporter.*

In connection with these cases, it may be mentioned that Dr. Linsly, of New York, has successfully used fumigations with Cinnabar in several cases of Diphtherite. —*Wood's Quarterly Retrospect.*

